# Circular RNA circFGFR1 Functions as an Oncogene in Glioblastoma Cells through Sponging to hsa-miR-224-5p

**DOI:** 10.1155/2022/7990251

**Published:** 2022-01-10

**Authors:** Qian Zhang, Shan Chen, Yingwei Zhen, Peng Gao, Zhenyu Zhang, Hao Guo, Yong Wang

**Affiliations:** ^1^Department of Oncology, The Central Hospital of Enshi Tujia and Miao Autonomous Prefecture, Hubei, China; ^2^Department of Neurosurgery, The First Affiliated Hospital of Zhengzhou University, Zhengzhou, Henan Province 450052, China; ^3^Department of Cardiology, The Central Hospital of Enshi Tujia and Miao Autonomous Prefecture, Hubei, China

## Abstract

Recently, increased studies have shown the important regulatory role of circular RNA (circRNA) in cancer progression and development, including glioblastoma (GBM). However, the function of circRNAs in glioblastoma is still largely unclear. Here, we state that circFGFR1 is elevated in glioma cells, resulting in aggravated glioma aggravated malignancy. The upregulation of circFGFR1 also promotes glioma growth in mouse xenograft models. Furthermore, CXCR4 level in glioma cells is positively correlated with circFGFR1 level, and higher CXCR4 expression is found in circFGFR1 overexpression groups. The effect of circFGFR1 on glioma malignancy is abolished in CXCR4 knockout cells. Then, RIP, RNA pull-down, and luciferase reporter assay results showed that hsa-miR-224-5p directly binds to circFGFR1 and CXCR4 mRNA. The CXCR4 3′-untranslated region (UTR) activated luciferase activity was reduced with hsa-miR-224-5p transfection, while it is reversed when cotransfected with circFGFR1, indicating that circFGFR1 acts as a hsa-miR-244-5p sponge to increase CXCR4 expression. The hsa-miR-224-5p expression is negatively corrected with the glioma malignancy through inhibiting CXCR4 level. Besides, the circFGFR1-induced regulation in glioma malignancy is also abrogated in hsa-miR-224-5p knockout cells. Taken together, our findings suggest that circFGFR1 plays a critical role in the tumorigenic behaviors in glioma cells by upregulating CXCR4 expression via sponging to hsa-miR-224-5p. These findings provide a new perspective on circRNAs during GBM development.

## 1. Introduction

Glioblastoma (GBM) is one of the most devastating cancers [1]. Despite recent advances in therapy, such as radiotherapy, chemotherapy, and mass surgical resection, the median survival is less than 15 months, and the 2-year survival rate is less than 5% [2, 3]. Thus, further understanding the potential molecular mechanisms in glioma progression is an emergency for exploring more effective therapeutic strategies.

Circular RNAs (circRNAs) are newly discovered noncoding RNAs generated from one or more exons [4]. Benefit from the covalently closed-loop structures without 5′ to 3′ polarity and polyadenylated tail [5], circRNAs are much more stable than linear RNAs in the cytoplasm, which might contribute to its significant role in different diseases and pathophysiological processes, including cancer progression [6–8]. Recently, Zhu et al. state that dysregulated circRNAs displayed a correlation with tumorigenesis and the development of GBM through bioinformatics, indicating the important role of circRNAs in glioma [9]. In addition, several reports demonstrated that circRNAs are involved in the glioma progression, such as circITGA7 [10], circSEPT9 [11], and circ-MAPK4 [12]. Hence, clarifying the molecular mechanisms induced by circRNAs in glioma may conduce to promising therapeutic candidate development.

Cell surface chemokine receptor (CXCR4) is involved in the proliferation, invasion, migration, angiogenesis, and metastasis in different tumors, including glioma [13, 14]. It is also clarified with heavily relating to the epithelial-mesenchymal transition and cancer stem cell survival in cancer cells [15]. Despite many reports focused on the molecular mechanism of CXCR4, the circRNA-induced CXCR4 regulation is still limited to be known in glioma.

The fibroblast growth factor receptor 1 (FGFR1) is a receptor tyrosine kinase that regulates the fibroblast growth factor signals [16], resulting in promoting the progression and development in different cancers, including glioma [17–19]. A higher level of FGFR1 is showed in GBM clinical samples from the GEPIA database, indicating that FGFR1-derived circRNA (circFGFR1) level might also be increased in glioma. Hence, we hypothesize that FGFR1-derived circRNAs may act as tumor promotors in glioma.

In this study, we found that a higher level of circFGFR1 (hsa_circ_0084003) was associated with the glioma malignant behavior involved in proliferation, migration, and invasion. Moreover, we found that this kind of oncogenicity was attributed to the increased CXCR4 expression induced by circFGFR1. We also found that hsa-miR-224-5p was responsible for the correlation between CXCR4 and circFGFR1. circFGFR1 could bind to hsa-miR-224-5p, acted as a microRNA (miRNA) sponge to increase the CXCR4 expression.

## 2. Methods

### 2.1. Cell Culture

The primary normal human astrocytes (NHA) and glioma cell lines (U251MG, U87MG, U118MG, T98G, SNB19) were purchased from American Type Culture Collection (ATCC) (Rockefeller, MD, USA). All cell lines were cultured with Dulbecco's Modified Eagle Medium (DMEM) (Gibco, Grand Island, NY) supplemented with 10% foetal bovine serum (FBS) (Gibco) and 100 U/mL penicillin and 100 *μ*g/mL streptomycin (Gibco) and incubated at 37°C, 5% CO_2_.

### 2.2. Gene Knockout Cell Lines Generated by Clustered Regularly Interspaced Short Palindromic Repeats (CRISPR)/Cas9 System

Cells with CXCR4 or hsa-miR-224-5p gene knockout were generated by the Cas9-GFP protein and sgRNA complex ribonucleoprotein (RNP). The Cas9-GFP and sgRNA were obtained from GenScript (Nanjing, Jiangsu, China). The experiment was performed as described in a previous study [20]. Briefly, SNB19 cells were plated into 6-well plates as 4 × 10^4^ cells per well 24 h before transfection. Next, OPTI-MEM mixed with Lipofectamine Cas9 Plus™ Reagent (Invitrogen, Carlsbad, CA, USA) was used to transfect Cas9/sgRNA RNPs into cells. 80 pmol Cas9-GFP protein and 80 pmol sgRNA were mixed to generate RNPs. The transfected cells were cultured for 3 days at 37°C, and single clones were generated with limiting dilution method in 96-well plates after sorting for GFP-positive cells using a FACSAria instrument (BD Biosciences, San Jose, CA, USA). The clones with successful CXCR4 or hsa-miR-224-5p knockout were identified by sequencing, RT-qPCR, and western blot assays and were used in the subsequent experiments. The sgRNA sequences were shown in Table S1.

### 2.3. Transduction and Transfection

Cells with stable overexpressing circFGFR1 or sh-circFGFR1 and their controls were generated by lentivirus transduction. Lentivirus containing circFGFR1 and sh-circFGFR1 targeting the junction region of the circFGFR1 sequence and Vehicle and sh-NC controls were synthesized by Hanbio Company (Shanghai, China) [17]. SNB19 cells were plated into 6-well plates at a cell density of 4 × 10^4^ cells/well 24 h before transduction. Then, cells were transduced with a 1 mL medium containing 25 *μ*L lentivirus (10 × 10^8^ TU/mL) and 8 *μ*g/mL polybrene. After 8 h, added 1 mL fresh medium and continuedly incubated cells at 37°C for another 24 h. Then, changed the medium with 4 *μ*g/mL puromycin to screen stable overexpressing cells. The expression of circFGFR1 was confirmed by qPCR.

miRNA negative control (mi-NC), miRNA mimics, miRNA inhibitor (inhibitor), and its negative control (inhibitor NC) were produced by Sangon Biotech (Shanghai, China). Plasmid for transiently expressing circFGFR1 (pCDNA3.1-circFGFR1) was obtained from Hanbio Company. Those oligonucleotides and plasmids were transfected into cells using Lipofectamine 3000 (Thermo Fisher, Grand Island, NY) following the manufacturer's instructions.

### 2.4. Quantitative Real-Time Polymerase Chain Reaction (RT-qPCR)

For circRNA detection, total RNA was extracted from cells by the RNeasy Protect Mini Kit (Qiagen), and then, linear RNA was digested using RNase R (Epicentre). Subsequently, RNA was reverse transcribed to cDNA by the QuantiNova Reverse Transcription Kit (Qiagen), and qPCR was performed with the TB Green® Advantage® qPCR Premix (TaKaRa, Japan). For mRNA detection, the steps were similar to circRNA detection except for linear RNA digestion.

For the detection of miRNA, miRNAs were extracted by using miRNeasy Micro Kit (Qiagen, German) and then reversed by using miScript II RT kit (Qiagen, German). The miRNAs quantify performed using miScript SYBR-Green PCR kit (Qiagen, German) with stem-loop qRT-PCR referring to others' work [21, 22]. GAPDH or U6 was used as the internal control. Relative expression of genes was measured by the 2-△△CT method. The sequences of the primers are displayed in Table S1.

### 2.5. Western Blotting

For western blot analysis, the total protein extracts from cells (RIPA, Beyotime Biotechnology, Shanghai, China) and tissue (One Step Animal Tissue Active Protein Extraction Kit, Sangon Biotech) were quantified by a bicinchoninic acid assay kit (Invitrogen). After that, proteins were separated by sodium dodecyl sulfate-polyacrylamide gel electrophoresis (SDS-PAGE) and then transferred onto polyvinylidene difluoride membranes. Then, the membranes were blocked with Tris-buffered saline supplementing with 0.1% Tween 20 (TBST) and 5% fat-free milk for 1 h and incubated with corresponding antibodies at 4°C overnight. The membranes were incubated with secondary antibodies at room temperature after washing with TBST 3 times. The membranes were measured by an image analysis system (Image-Pro Plus 6.0, Media Cybernetics, Rockville, MD, USA) after incubating with a high-signal electrochemiluminescence kit (Fdbio Science, Hangzhou, China).

### 2.6. RNA Immunoprecipitation (RIP)

Magna RIP™ RNA-Binding Protein Immunoprecipitation kit (Millipore, Billerica, MA, USA) was used to conduct RIP assay according to the manufacturer's instructions. Briefly, 100 *μ*L RIP Lysis Buffer was used to resuspend approximately 5 × 10^6^ cells containing protease and RNase inhibitors. Then, cell lysates were incubated with IgG isotype (1 : 150) or anti-AGO2 antibody (1 : 50) (Abcam, Hangzhou, China) mixed with magnetic beads at 4°C overnight, respectively. After treating with proteinase K, the immunoprecipitated RNAs were extracted by phenol-chloroform extraction. The enriched RNAs were measured by qRT-PCR, as mentioned above.

### 2.7. CircRNAs In Vivo Precipitation (circRIP)

CircRIP assays were performed as others work [17]. Briefly, cells with circFGFR1 overexpression were fixed with 1% formaldehyde for 30 min after washing with ice-cold phosphate-buffered saline (PBS). Then, cells were lysed in 500 *μ*L co-IP buffer, sonicated and centrifuged. After that, a probes-M280 streptavidin dynabeads (Invitrogen) mixture was added into the supernatant mixed with a circFGFR1-specific probe and incubated at 30°C for 12 h. After that, the probes-dynabeads-circRNAs mixture was incubated with 200 *μ*L lysis buffer and proteinase K. Subsequently, the RNA was extracted from the mixture using phenol-chloroform extraction and analyzed by qPCR.

### 2.8. RNA Pull-Down

RNA pull-down assays were performed as described previously [23]. Briefly, cell extract was mixed with biotinylated RNA. Washed streptavidin agarose beads were added to each binding reaction and incubated at room temperature for 1 h. Beads were washed briefly three times, and the coprecipitated RNAs were detected by q-PCR. Controls were also assayed to demonstrate that the detected signals were reliable.

### 2.9. Luciferase Reporter Assay

The predicted sequences of circRNA or mRNA were inserted into pGL4-luciferase reporter plasmids (Promega, Madison, USA). The reporter plasmids and miRNAs were cotransfected into cells using Lipofectamine 3000 (Life Technologies). The dual-luciferase reporter assay system (Promega) was used to measure the luciferase activity in glioma cells after transfection for 48 h.

### 2.10. Matrigel-Based or Matrigel-Free Transwell Assay

Matrigel-based Transwell assay was used to measure the invasion of glioma cells, and Matrigel-free-based Transwell assay was used to detect the migration of cells. For Matrigel-based Transwell assay, a 24-well insert with an 8 *μ*m pore size was coated with Matrigel (Corning, Shanghai, China) at 37°C for 1 h according to the manufacturer's directions. Then, 3 × 10^5^ cells in 250 *μ*L FBS-free medium were plated into the upper side of the Transwell chamber by adding 0.5 mL medium with 30% FBS to the lower side. 48 h later, cells on the underside of the membrane were fixed and stained and further counted in 5 random fields under a microscope. For Matrigel-free Transwell assay, the steps were similar to Matrigel-based assay except for coating Matrigel.

### 2.11. Cell Proliferation Assay

Cell proliferation was tested using Cell Counting Kit 8 (CCK8, Beyotime, Shanghai, China). Cells were seeded into 96-well plates at 3000 cells/well and cultured for 0 hours, 24 hours, 48 hours, 72 hours, 96 hours, and 120 hours. The CCK8 solution was added and incubated for 2 hours. The absorbance was measured at 450 nm.

### 2.12. Xenograft Model

BALB/c nude mice (6 weeks old) were purchased from Vital River Laboratory Animal Technology Co., Ltd. (Beijing, China). After adopting the new environment for one week, mice were randomly divided into 2 groups (*n* = 6). Engineered SNB19 cells (1 × 10^7^) with circFGFR1, sh-circFGFR1, or their corresponding control overexpression were subcutaneously injected into the back region of BALB/c nude mice. The tumor volume was measured every 5 days after injection using a Vernier caliper with volume = 0.5 × length × width. Tumor weight was determined after 30 days.

### 2.13. Statistics Analysis

All quantitative data were evaluated to determine the normality of the distribution using the Shapiro-Wilk test. The student's *t*-test was performed using Prism (version 5; GraphPad Software), and one-way ANOVA followed by Tukey's multiple comparison test was used to analyze multiple groups. Data are expressed as the mean ± SD. Values were considered statistically significant at *p* < 0.05.

## 3. Results

### 3.1. Upregulation of circFGFR1 Is Associated with Glioma Development

**Figure 1:**
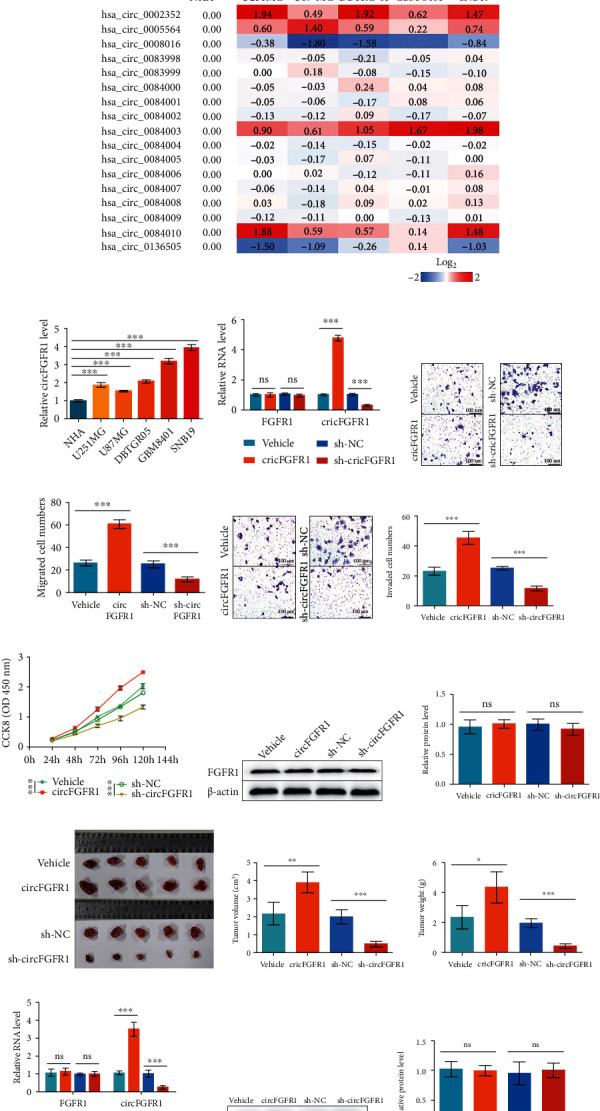
Upregulation of circFGFR1 promotes the glioma development. (a) The clinical data of FGFR1 expression in the normal persons and GBM patients from GEPIA database. (b) The heat map of circRNA expression in different cell lines analyzed by qPCR. Data are processed with Log2. (c) The expression profile of circFGFR1 in different cell lines from (b). (d–k) SNB19 cells overexpressing Vehicle, circFGFR1, sh-NC, and sh-circFGFR1, respectively, by transducing lentivirus and screening with puromycin. (d) The expression of FGFR mRNA and circFGFR1 in those engineered SNB19 stable cell lines analyzed by qPCR. (e, f) The migration ability of those engineered SNB19 stable cell lines analyzed by Matrigel-free Transwell assay. (g, h) The invasion ability of those engineered SNB19 stable cell lines analyzed by Matrigel-based Transwell assay. (i) The proliferation ability of engineered SNB19 cell lines analyzed by CCK8 assay. (j, k) The expression of FGFR1 protein in engineered SNB19 cell lines analyzed by western blot. (l–q) The xenograft tumors generated by injecting SNB19 cells stable overexpressing Vehicle, circFGFR1, sh-NC, and sh-circFGFR1 into nude mice, respectively. (l) The images of xenograft tumor in different groups. (m, n) The tumor volume and weight at 30 days after cell injection. (o) The expression of FGFR1 mRNA and circFGFR1 in xenograft tumors analyzed by qPCR. (p, q) The expression of FGFR1 protein in xenograft tumors analyzed by western blot. The data are presented as the means ± SD, *n* = 3 experiments in (b–k), *n* = 6 experiments in (l–q), ^∗^*p* < 0.05, ^∗∗^*p* < 0.01, ^∗∗∗^*p* < 0.005.

In terms of the critical roles of the FGFR1 in GBM, we analyzed its expression in clinical samples from the GEPIA database (http://gepia.cancer-pku.cn/index.html). The FGFR1 level in GBM patients was more than 2-fold higher than it is a normal person (Figure 1(a)), indicating a possibility of increased FGFR1-derived circRNAs in glioma. Thus, we found a total of 17 FGFR1-derived circRNAs through the CircInteractome database (https://circinteractome.nia.nih.gov/). Those circRNAs' expression was measured by qPCR in one primary NHA cell and six glioma cell lines (U251MG, U87MG, DBTRG-05, GBM 8401, SNB19) (Figure 1(b)). Interestingly, 4 of them were upregulated (hsa_circ_0002352, hsa_circ_0005564, hsa_circ_00084003, and hsa_circ_00084010) while 2 of them were downregulated (hsa_circ_0008016 and hsa_circ_0136505). Those changed circRNAs were already reported in glioma cell lines or central nervous system-related cells [24–26], excepting hsa_circ_00084003, only found in lung-related cells [17]. Hsa_circ_00084003, named as circFGFR1 by Zhang et al., has been shown a promotive activity in the progression and anti-PD-1 resistance in NSCLC (Figure S1) [17]. However, its function in glioma is unclear. Therefore, we want to understand whether circFGFR1 also significantly influences glioma development, like it in NSCLC (the circFGFR1 expression in NHA and other six glioma cell lines are shown in Figure 1(c)).

For that reason, we, at first, investigate the effect of circFGFR1 on glioma malignancy. We used the SNB19 cell line as a cell model because of the highest circFGFR1 expression. We generated SNB19 cells with circFGFR1 or sh-circFGFR1 stable overexpression by transducing with lentivirus and selecting with puromycin. The circFGFR1 expressions in engineered SNB19 cells are showed in Figure 1(d), indicating successful cell line construction. The results of the Transwell assay without coating Matrigel, reflecting the migration ability of cells, showed that enhanced cell numbers were observed in SNB19 cells with circFGFR1 overexpression comparing with controls. In contrast, the cell numbers were decreased in the sh-circFGFR1 overexpression group (Figures 1(e) and 1(f)). Additionally, the invasion ability of SNB19 cells was detected by Transwell assay with coating Matrigel. Moreover, similar results were monitored. The cell numbers in circFGFR1 overexpression cells were higher than controls, which was reduced in the sh-circFGFR1 group (Figures 1(g) and 1(h)). Also, we measured the proliferation of SNB19 cells by using the CCK8 assay. A promoted proliferation was displayed in SNB19 cell overexpressing circFGFR1 with the inhibition in the sh-circFGFR1 group (Figure 1(i)). Those results suggested that increased circFGFR1 was relevant with the deteriorative invasion and migration ability and activated proliferation in SNB19 cells. We also found that FGFR1 expression was not affected by circFGFR1 comparing with different engineered SNB19 cell lines (Figures 1(d), 1(j), and 1(k)), indicating that the malignancy change induced by circFGFR1 in SNB19 cell was independent with FGFR1.

For confirming the role of circFGFR1, we performed a mouse xenograft model to investigate its role in glioma development in vivo. SNB19 cells with stably overexpressing circFGFR1 or sh-circFGFR1 were subcutaneously injected into nude mice. The group with circFGFR1 overexpression showed an accelerated tumor growth compared with control, but the tumor growth was inhibited in the sh-circFGFR1 group, including the tumor volume and weight (Figures 1(l)–1(n)). Then, the invariant FGFR1 expression was confirmed in tumors using qPCR and western blot to exclude the FGFR1 effects on glioma development (Figures 1(o)–1(q)).

Together, those above findings suggested that circFGFR1 could promote the malignancy and development of glioma cells in vitro and in vivo.

### 3.2. CXCR4 Is Responsible for the circFGFR1-Induced Exasperated Glioma Progression

**Figure 2:**
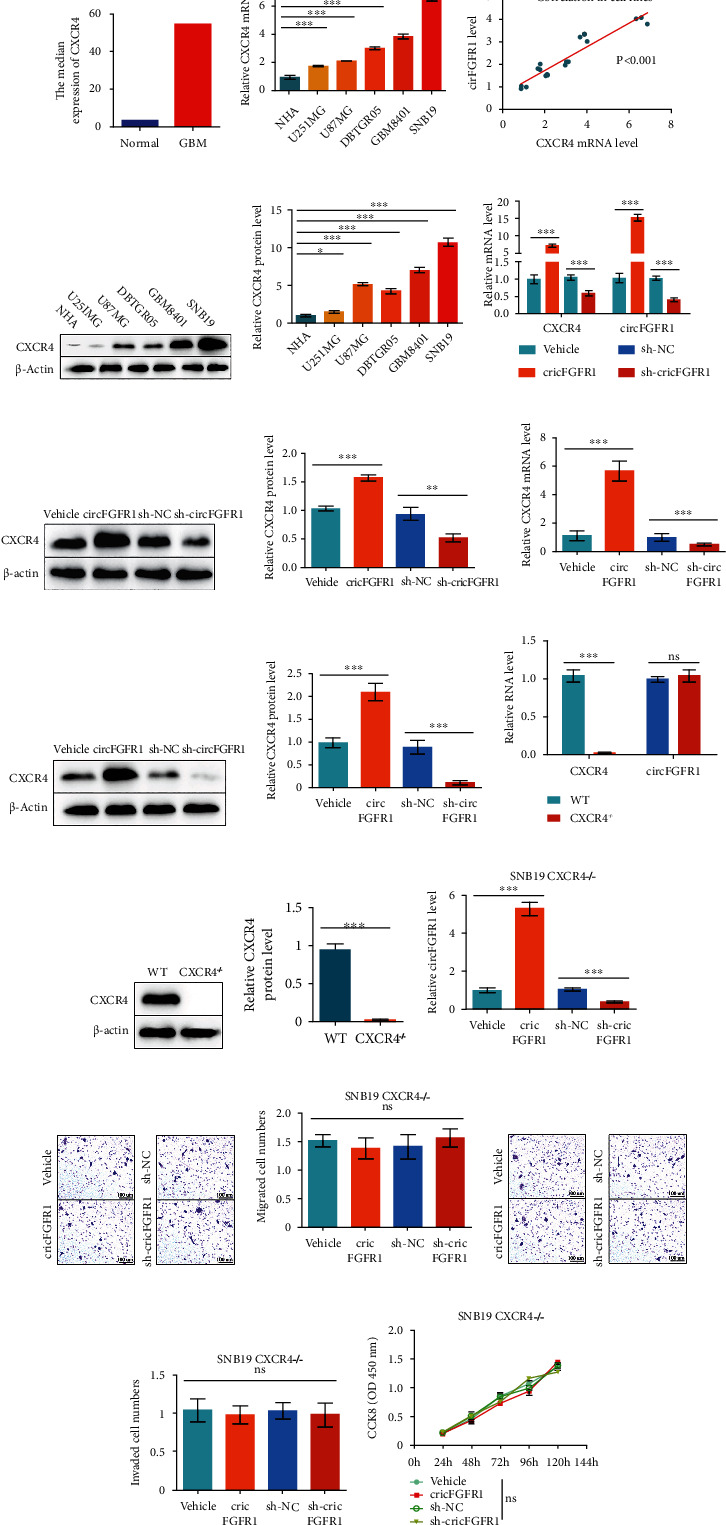
The circFGFR1-induced exasperated glioma progression is attributed to CXCR4 upregulation. (a) The clinical data of CXCR4 expression in the normal persons and GBM patients from the GEPIA Database. (b) The expression profile of CXCR4 mRNA in different cell lines and NHA analyzed by qPCR. (c) The correlation of CXCR4 mRNA level and circFGFR1 level in different cell lines and NHA. (d, e) The expression of CXCR4 protein in different cell lines and NHA analyzed by western blot. (f–l) SNB19 cells overexpressing Vehicle, circFGFR1, sh-NC, and sh-circFGFR1, respectively, by transducing lentivirus and screening with puromycin. (f) The expression of CXCR4 mRNA in engineered SNB19 stable cell lines analyzed by qPCR. (g, h) The expression of CXCR4 protein in engineered SNB19 stable cell lines analyzed by western blot. (i) The expression of CXCR4 mRNA in xenograft tumors analyzed by qPCR. (j, k) The expression of CXCR4 protein in xenograft tumors analyzed by western blot. (l) The expression of CXCR4 mRNA and circFGFR1 in WT SNB19 cells and SNB19^CXCR4-/-^ cells analyzed by qPCR. (m, n) The expression of CXCR4 protein in WT SNB19 cells and SNB19^CXCR4-/-^ cells analyzed by western blot. (o–t) SNB19^CXCR4-/-^ cells overexpressing Vehicle, circFGFR1, sh-NC, and sh-circFGFR1, respectively, by transducing lentivirus and screening with puromycin. (o) The expression of circFGFR1 in engineered SNB19^CXCR4-/-^ stable cell lines analyzed by qPCR. (p, q) The migration ability of those engineered SNB19^CXCR4-/-^ stable cell lines analyzed by Matrigel-free Transwell assay. (r, s) The invasion ability of those engineered SNB19^CXCR4-/-^ stable cell lines analyzed by Matrigel-based Transwell assay. (t) The proliferation ability of engineered SNB19CXCR4-/- cell lines analyzed by CCK8 assay. The data are presented as the means ± SD, *n* = 3 experiments in (b–h) and (l–t), *n* = 6 experiments in (i–k), ^∗^*p* < 0.05, ^∗∗^*p* < 0.01, ^∗∗∗^*p* < 0.005.

Inspired by Zhang et al.'s work [17], we supposed whether the circFGFR1 could regulate CXCR4 in glioma cells, which might be the reason for the changed glioma malignant behaviors. Thus, we analyzed the clinical data from the GEPIA database to verify the CXCR4 level in GBM. An extremely higher expression of CXCR4 was found in GBM from patients compared with the normal persons (Figure 2(a)). Then, we analyzed CXCR4 expression in NHA and other glioma cell lines by qPCR (Figure 2(b)). It was easily found that the level of CXCR4 in glioma cells was higher than NHA cells, and the abundance of CXCR4 was positively correlated with the circFGFR1 level in glioma cell lines (Figure 2(c)). The protein level of CXCR4 was also detected in NHA and glioma cell lines, consistent with the qPCR results (Figures 2(d) and 2(e)). Further, we found increased CXCR4 level in SNB19 cells with circFGFR1 overexpression and a downregulated level in the sh-circFGFR1 group, both in mRNA and protein levels (Figures 2(f)–2(h)). We also found similar results in xenograft tumors (Figures 2(i)–2(k)).

In order to further confirm whether CXCR4 is responsible for the circFGFR1-induced glioma growth change, we generated a CXCR4 knockout SNB19 cell line by using CRISPR/Cas9 with a ribonucleoprotein (RNP) system. The CXCR4 level was confirmed by qPCR and western blot in SNB19^CXCR4-/-^ cells (Figures 2(l)–2(n)). Using the SNB19^CXCR4-/-^ cells, we transduced circFGFR1 or sh-circFGFR1 into SNB19^CXCR4-/-^ cells for generating stable overexpression cell lines. The circFGFR1 level in engineered SNB19^CXCR4-/-^ cell lines was identified by qPCR (Figure 2(o)). The results of Transwell assays and CCK8 assay showed no significant change between SNB19^CXCR4-/-^-based engineered cell lines (Figures 2(p)–2(t)), indicating that the invasion, migration, and proliferation were irrelevant with circFGFR1 in CXCR4 defected SNB19 cells.

Together, those findings stated that CXCR4 was obbligato and responsible for circFGFR1-induced glioma progression regulation.

### 3.3. circFGFR1 Acts as a hsa-miR-224-5p Sponge for Regulating CXCR4 Expression

**Figure 3:**
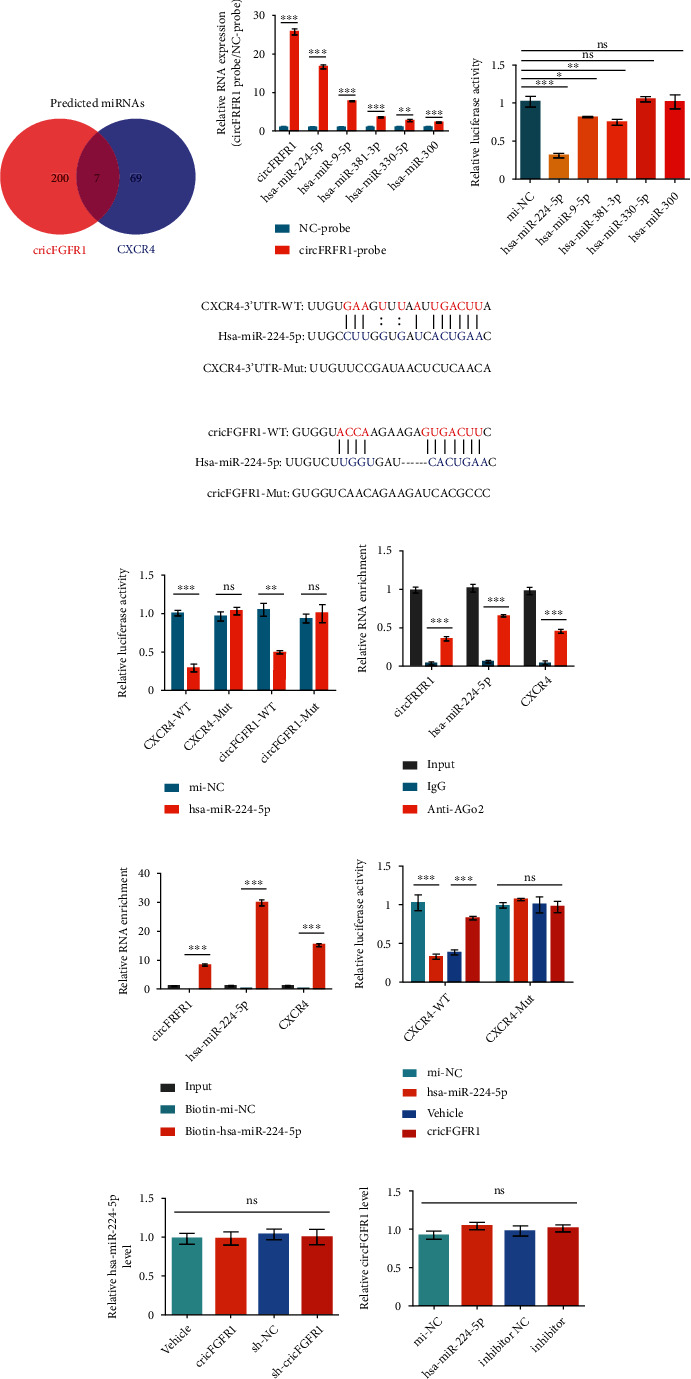
circFGFR1 increases CXCR4 level through sponging to hsa-miR-224-5p. (a) The intersection of predicted miRNA targeting circFGFR1 and CXCR4 according to Starbase database. (b) miRNAs binding with circFGFR1 in SNB19 cells analyzed by circRIP assay. (c) The luciferase activity of pLG3-circFGFR1 in the SNB19 cells after cotransfection with miRNAs. (d) Putative binding sites of hsa-miR-224-5p with respect to circFGFR1 and CXCR4 3′-URT were predicated via Starbase. (e) The luciferase activity of pLG3-circFGFR1 or pLG3-CXCR4 3′-UTR in the SNB19 cells after cotransfection with hsa-miR-224-5p. (f) The RNA enrichment of circFGFR1, hsa-miR-224-5p, and CXCR4 in SNB19 cells after immunoprecipitated with anti-AGO2 antibody using RIP assay. (g) The RNA enrichment of circFGFR1, hsa-miR-224-5p, and CXCR4 in SNB19 cells using RNA pull-down assay. SNB19 cells were transfected with biotin-labeled mi-NC or hsa-miR-225-5p and pull down with streptavidin agarose beads. (h) The luciferase activity of pLG3- CXCR4 3′-UTR in the SNB19 cells after cotransfecting with hsa-miR-224-5p solely or combined with pCDNA3.1-circFGFR1. (i) The expression of hsa-miR-224-5p in SNB19 cells stable overexpressing Vehicle, circFGFR1, sh-NC, and sh-circFGFR1, respectively, using qPCR. (j) The expression of circFGFR1 in SNB19 cells transfected with mi-NC, hsa-miR-224-5p, inhibitor NC, and inhibitor, respectively, using qPCR. The data are presented as the means ± SD, *n* = 3 experiments in (b–j), ^∗^*p* < 0.05, ^∗∗^*p* < 0.01, ^∗∗∗^*p* < 0.005.

For exploring the molecular mechanism of CXCR4 regulation induced by circFGFR1, which might be attributed to miRNA, we made an intersection with those predicted miRNAs binding with CXCR4 mRNA and circFGFR1, respectively. As a result, there were only 7 miRNAs in the intersection (Figure 3(a) and Table 1). Then, we evaluate the importance of those miRNAs by analyzing the *p* value of the clinical survival curve in GBM (data from Starbase database, http://starbase.sysu.edu.cn/index.php) (Table 1). After excluding the miRNAs with *p* value > 0.5 in the survival curve, there were 5 miRNAs left; one of them is hsa-miR-381-3p, the miRNA contributing to circFGFR1-induced CXCR4 regulation in NSCLC [17]. Considering the difference between GBM and NSCLC, we analyzed the binding ability of those 5 miRNAs with circFGFR1 in SNB19 cells by using circRIP assay. We used a cirFGFR1-specific probe to immunoprecipitate circFGFR1 by dynabeads and then tested by the qPCR for measuring the potential miRNAs in the complex. Difference with it in A549 cells [17], we found that hsa-miR-224-5p displayed the highest enrichment among those 5 miRNAs, indicating its strongest binding ability with circFGFR1 in SNB19 cells (Figure 3(b)). Next, we performed a luciferase activity assay in SNB19 cells to confirm the circRIP results. Hsa-miR-224-5p showed the most reduction of luciferase activity comparing with others (Figure 3(c)). For further verifying the results, we tested the binding ability between hsa-miR-224-5p and CXCR4 or circFGFR1 by using luciferase activity in SNB19 cells. The results showed the reduced luciferase activity in wide-type CXCR4 and circFGFR1 but not in mutant groups, indicating that hsa-miR-224-5p could directly bind to the 3′-untranslated regions (UTR) of CXCR4 and circFGFR1 (Figures 3(d) and 3(e)).

Further, we explored the interaction between CXCR4 mRNA, hsa-miR-224-5p, and circFGFR1. The RIP results displayed a coenrichment of CXCR4 mRNA, hsa-miR-224-5p, and circFGFR1 (Figure 3(f)). The results of RNA pull-down immunoprecipitated with biotin-labeled hsa-miR-224-5p showed an obvious enrichment of circFGFR1 and CXCR4 mRNA (Figure 3(g)). Those results indicated that hsa-miR-224-5p directly interacted with CXCR4 mRNA and circFGFR1 simultaneously. Moreover, we found that the reduced luciferase activity cotransfected with hsa-miR-224-5p mimics and pGL3-LUC-CXCR4 (wide type (WT)) plasmid were revised in the presence of wide type circFGFR1 expression plasmid, but not in mutant (Mut) groups (Figure 3(h)). Besides, the hsa-miR-224-5p expression was no changes in SNB19 cells with circFGFR1 overexpression and downregulated in overexpressing sh-circFGFR1 SNB19 cells, and the circFGFR1 level showed no significant difference after the hsa-miR-224-5p expression was either increased or knocked down in SNB19 cells (Figures 3(i) and 3(j)), indicating that circFGFR1 and hsa-miR-224-5p were not degraded by each other.

Together, those findings demonstrated that circFGFR1 regulates CXCR4 expression through the sponge to hsa-miR-224-5p.

### 3.4. Sponging to hsa-miR-224-5p Induced by circFGFR1 Contributes to Glioma Oncogenicity

**Figure 4:**
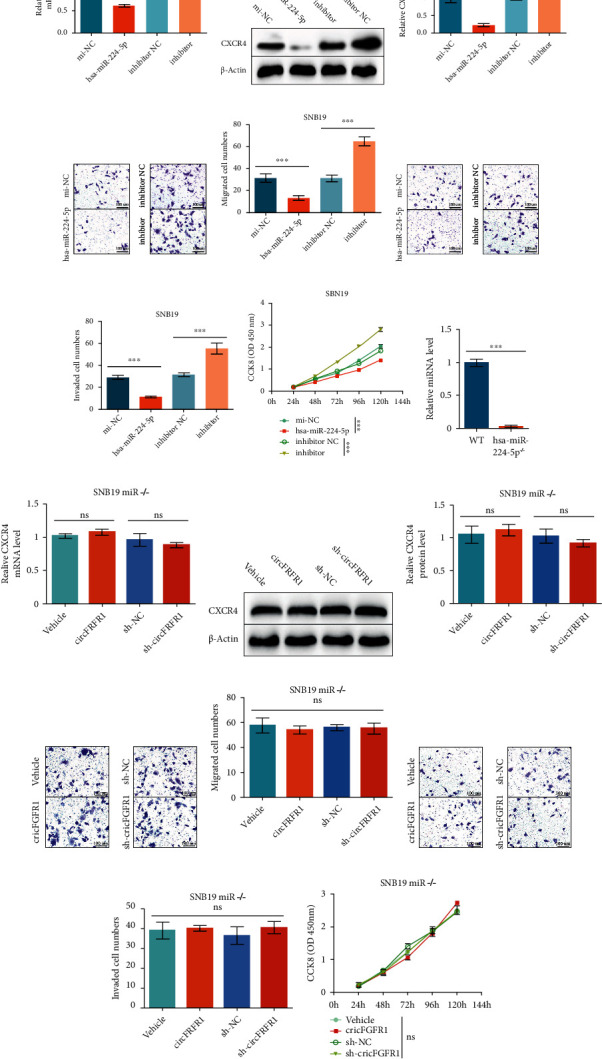
Hsa-miR-224-5p is necessary for circFGFR1-induced glioma oncogenicity. (a–h) SNB19 cells transfected with mi-NC, hsa-miR-224-5p, inhibitor NC, and inhibitor, respectively. (a) The expression of CXCR4 mRNA in those different groups analyzed by qPCR. (b, c) The expression of CXCR4 protein in those different groups analyzed by western blot. (d, e) The migration ability of those different groups analyzed by Matrigel-free Transwell assay. (f, g) The invasion ability of those different groups analyzed by Matrigel-based Transwell assay. (h) The proliferation ability of those different groups analyzed by CCK8 assay. (i) The expression of hsa-miR-224-5p in WT SNB19 cells and SNB19^miR-/-^ cells analyzed by qPCR. (j–q) SNB19^miR-/-^ cells overexpressing Vehicle, circFGFR1, sh-NC, and sh-circFGFR1, respectively, by transducing lentivirus and screening with puromycin. (j) The expression of CXCR4 mRNA in engineered SNB19^miR-/-^ stable cell lines analyzed by qPCR. (k, l) The expression of CXCR4 protein in engineered SNB19^miR-/-^ stable cell lines analyzed by western blot. (m, n) The migration ability of t engineered SNB19^miR-/-^ stable cell lines analyzed by Matrigel-free Transwell assay. (o, p) The invasion ability of engineered SNB19^miR-/-^ stable cell lines analyzed by Matrigel-based Transwell assay. (q) The proliferation ability of engineered SNB19^miR-/-^ stable cell lines analyzed by CCK8 assay. The data are presented as the means ± SD, *n* = 3 experiments in (a–q), ^∗^*p* < 0.05, ^∗∗^*p* < 0.01, ^∗∗∗^*p* < 0.005.

We then further investigate whether the circFGFR1 function as sponging to hsa-miR-224-5p is the reason for exasperate glioma biobehaviors. For that purpose, we, firstly, detected the CXCR4 expression in SNB19 cells with increased hsa-miR-224-5p expression or knocked down. The mRNA and protein levels of CXCR4 were significantly reduced with hsa-miR-224-5p transfection, accompanying the inhibited invasion, migration, and proliferation of SNB19 cells (Figures 4(a)–4(h)). On the other hand, the suppression of hsa-miR-224-5p in SNB19 cells induced by its inhibitor triggered the upregulation of CXCR4 expression, as well as promoted invasion, migration, and proliferation of glioma cells (Figures 4(a)–4(h)). These results implied that inhibited hsa-miR-224-5p function was contributed to the glioma oncogenicity by increasing the CXCR4 level.

To further confirm the role of hsa-miR-224-5p in circFGFR1-induced CXCR4 regulation and glioma progression, we generated hsa-miR-224-5p knockout SNB19 cell line by using CRISPR/Cas9 with RNP system. Then, SNB19^miR-/-^ overexpressing circFGFR1 or sh-circFGFR1 and their controls were constructed by transducing lentivirus and selecting with puromycin. The abundance of hsa-miR-224-5p was determined by qPCR, verifying the miRNA knockout (Figure 4(i)). Interestingly, we found that both mRNA and protein levels of CXCR4 showed no significant changes in cells with circFGFR1 upregulation or knockdown after hsa-miR-224-5p knockout (Figures 4(j)–4(l)), different from the results before miRNA knockout (Figures 2(f)–2(h)). Moreover, the invasion and migration, measured by Transwell assay, and proliferation, tested by CCK8 assay, were also independent with circFGFR1 level in SNB19^miR-/-^ cells (Figures 4(m)–4(q)), completely different from previous results (Figures 1(e)–1(i)).

Together, our findings hinted that the circFGFR1-induced hsa-miR-224-5p sponge was the culprit of elevated CXCR4 abundance and aggravated malignant behaviors in glioma cells. Besides, hsa-miR-224-5p could downregulate CXCR4 level in glioma cells, resulting in the inhibited glioma development.

## 4. Discussion

Despite considerable effort, with much of the perceived improvement in two prognostic biomarkers, mutations in isocitrate dehydrogenase (IDH) and O6-methylguanine-methyltransferase (MGMT) promoter methylation [27], little progress has been made toward prolonged survival in GBM. On the other hand, although many strategies have been performed in GBM therapy, such as surgery, radiation therapy, chemotherapy, and drugs targeting many different molecules, kinases, or proteins [28], the survival performance in GBM is still poor with the 2-year survival rate less than 5% [2, 3]. Thus, further understanding of the pathogenic mechanism in GBM is essential to find more specific biomarkers for diagnosis or more effective targets for drug development.

Recently, an increasing number of reports demonstrated the importance of noncoding RNAs, including circRNAs, miRNAs, and long noncoding RNAs (lncRNAs), in GBM progression [10–12, 29–31]. However, comparing with miRNA and lncRNA reports, much fewer studies were relevant on circRNAs in glioma (only 194 results about circRNA in glioma in PubMed, comparing with 1040 results in lncRNA and 2835 results in miRNA). Notably, none of any studies were relevant with circRNA-induced CXCR4 regulation in glioma, even with the nonnegligible CXCR4 function in glioma. Here, we, for the first time, identify that a circFGFR1 is a critical circRNA associated with the CXCR4 expression and glioma oncogenicity. We find that circFGFR1is upregulated in glioma cells as an oncogene to promote glioma progression in vitro and in vivo. What is more, circFGFR1 could act as a sponge of hsa-miR-224-5p to increase CXCFR4 level in glioma cells, thereby promoting the malignant behaviors of glioma cells, including in invasion, migration, and proliferation.

CircRNA always worked as miRNA sponges or competitors of endogenous RNAs (ceRNAs) to trigger the dysregulation of functional miRNAs and their target genes, with the results of tumor invasion, migration, and proliferation in cancers, including glioma [32, 33]. For instance, circITGA7 accelerates glioma progression via miR-34a-5p/VEGFA axis [10]; circSEPT9 promotes the malignant behaviors in glioma cells via miR-432-5p-mediated regulation of LASP1 [11]. On the other hand, circRNAs also act as inhibitors in glioma development, such as circCDR1as inhibiting gliomagenesis by disrupting the p53/MDM2 complex [33]. Our findings, first time, identify that circFGFR1 promotes glioma malignancy through hsa-miR-5p/CXCR4 signaling. Unlike the mechanisms of circRNAs as miRNA sponges, including our findings, they could also bind with proteins directly. For example, circSMARCA5, a downregulated tumor suppressor in glioblastoma, binds with SRSF1 directly via the GAUGAA motif and acts as a decoy for the oncogenic SRSF1, resulting in the suppression of SRSF1-induced oncogenicity [34, 35].

In fact, hsa-miR-224-5p has been reported to suppress tumor development in uveal melanoma [36]. At the same time, it also has proved to promote tumor progression in papillary thyroid carcinoma [37] and cystadenocarcinoma [38] or to promote cell survival in breast cancer [39]. Furthermore, hsa-miR-224-5p could be sponged by lncRNA and circRNAs in difference cancers, for instance, by lncNEAT1 in melanoma to promote tumor development [40] or by lncRNA MIR503HG in triple-negative breast cancer to prevent tumor progression [41] or by hsa_circ_0017639 in gastric cancer as a promotor [42] or circ-ITCH in hepatocellular carcinoma as a suppressor [43]. Notably, Zheng et al. stated that circPCMTD1 could sponge to hsa-miR-224-5p to promote glioma progression [44]. Those different regulatory mechanisms of hsa-miR-224-5p indicated complicated roles and multiple possibilities in miRNA sponge signaling with different tissues or cells. Although Zhang et al. have proved that circFGFR1 regulates CXCR4 expression in NSCLC, the miRNA between circFGFR1 and CXCR4 is different from our findings. We found that circFGFR1 regulates CXCR4 expression in glioma cells through sponging to hsa-miR-224-5p, which is hsa-miR-381-3p in NSCLC cell according to Zhang et al.'s work [17], further reflecting the cell or tissue-specific interaction between circRNAs and miRNAs.

Also, there are several defects in this study. On the one hand, we lack the clinical samples of GBM to confirm the circFGFR1 expression and the correlation between CXCR4 level and circFGFR1 level in clinical samples. On the other hand, the regulation of circFGFR1 needs to be further investigated for the possibility of developing circFGFR1 as a therapeutic target in the future.

## 5. Conclusion

Taken together, our results demonstrate that circFGFR1 expression is upregulated in glioma cells, and the elevated circFGFR1 level is responsible for the exasperating malignant behaviors in glioma cells, including invasion, migration, and proliferation, via increasing CXCR4 level, a critical oncogene in several cancers, by sponging to hsa-miR-224-5p. Hence, suppression of circFGFR1-induced hsa-miR-224-5p/CXCR4 signaling may be a potential therapeutic strategy in GBM.

## Figures and Tables

**Table 1:** miRNA's value of overall survival in brain lower grade glioma (LGG) cancer.

miRNA ID	miRNA name	*p* value of overall survival in LGG cancer
MIMAT0000281	hsa-miR-224-5p	9.4*E*-5
MIMAT0000441	hsa-miR-9-5p	2*E*-7
MIMAT0000736	hsa-miR-381-3p	8.8*E*-4
MIMAT0000756	hsa-miR-326	0.11
MIMAT0004693	hsa-miR-330-5p	0.0039
MIMAT0004903	hsa-miR-300	5.8*E*-10
MIMAT0019853	hsa-miR-4731-5p	0.88

## Data Availability

The data used to support the findings of this study are available from the corresponding author upon request.

## Abbreviations

CircRNA: Circular RNA
GBM: Glioblastoma
NSCLC: Non-small-cell lung cancer
CXCR4: Cell surface chemokine receptor
FGFR1: Fibroblast growth factor receptor 1
NHA: Normal human astrocytes
ATCC: American type culture collection
DMEM: Dulbecco's Modified Eagle Medium
FBS: Foetal bovine serum
CRISPR: Clustered regularly interspaced short palindromic repeats
RNP: Ribonucleoprotein
RT-qPCR: Real-time polymerase chain reaction
SDS-PAGE: Sodium dodecyl sulfate-polyacrylamide gel electrophoresis
TBST: Tris-buffered saline supplementing with 0.1% Tween 20
RIP: RNA immunoprecipitation
circRIP: circRNAs in vivo precipitation
CCK8: Cell Counting Kit 8
lncRNAs: Long noncoding RNAs
UTR: Untranslated regions
ceRNAs: Competitors of endogenous RNAs
WT: Wide type
Mut: Mutant.

## Supplementary Materials

Supplementary 1 Table S1: primers and sequences.

Supplementary 2 Figure S1: the scheme illustrating the production of circFGFR1.
