# Supplementary material for: Circular RNA circFGFR1 Functions as an Oncogene in Glioblastoma Cells through Sponging to hsa-miR-224-5p
Source: J Immunol Res. 2022 Jan 10;2022:7990251. doi: 10.1155/2022/7990251 (PMC8764274; doi:10.1155/2022/7990251)
Supplement: Supplementary 1 — Table S1: primers and sequences. [file 7990251.f1.docx]

Table S1. Primers and sequences.

| Name | Application | Primer 5’-3’ |
| --- | --- | --- |
| hsa_circ_0002352-F | qPCR | TGGAAGTGCCTCCTCTTCTG |
| hsa_circ_0002352-R |  | GGTGACAAGGCTCCACATCT |
| hsa_circ_0005564-F |  | CACATCCAGTGGCTAAAGCA |
| hsa_circ_0005564-R |  | GCAGGTGTAGTTGCCCTTGT |
| hsa_circ_0008016-F |  | AGACTCCGGCCTCTATGCTT |
| hsa_circ_0008016-R |  | GACCAGGAAGGACTCCACTTC |
| hsa_circ_0083998-F |  | ACAGAGACCCACCTTCAAGC |
| hsa_circ_0083998-R |  | CACATCACTCTGGTGGGTGT |
| hsa_circ_0083999-F |  | AGAGACCCACCTTCAAGCAG |
| hsa_circ_0083999-R |  | CGAGGCCAAAGTCTGCTATC |
| hsa_circ_0084000-F |  | TCACAGAGACCCACCTTCAA |
| hsa_circ_0084000-R |  | TGGGGTTGTAGCAGTATTCCA |
| hsa_circ_0084001-F |  | CCTCACAGAGACCCACCTTC |
| hsa_circ_0084001-R |  | CACATGAACTCCACGTTGCT |
| hsa_circ_0084002-F |  | CCTCACAGAGACCCACCTTC |
| hsa_circ_0084002-R |  | GCAGGTGTAGTTGCCCTTGT |
| hsa_circ_0084003-F |  | TCACAGAGACCCACCTTCAA |
| hsa_circ_0084003-R |  | TGCATGCAATTTCTTTTCCA |
| hsa_circ_0084004-F |  | ACAGAGACCCACCTTCAAGC |
| hsa_circ_0084004-R |  | TCCAACATACAGGGTGGACA |
| hsa_circ_0084005-F |  | GGAGTATCTGGCCTCCAAGA |
| hsa_circ_0084005-R |  | CAGCTCCACATCCCAGTTCT |
| hsa_circ_0084006-F |  | GTGCTTGGCGGGTAACTCTA |
| hsa_circ_0084006-R |  | TGATGCTGCCGTACTCATTC |
| hsa_circ_0084007-F |  | CACATCCAGTGGCTAAAGCA |
| hsa_circ_0084007-R |  | CAGCTCCACATCCCAGTTCT |
| hsa_circ_0084008-F |  | ACTCTGTGGTGCCCTCTGAC |
| hsa_circ_0084008-R |  | CAGGAAGGACTCCACTTCCA |
| hsa_circ_0084009-F |  | GAAGTTCAAATGCCCTTCCA |
| hsa_circ_0084009-R |  | ACAAGGCTCCACATCTCCAT |
| hsa_circ_0084010-F |  | CTGGTCACAGCCACACTCTG |
| hsa_circ_0084010-R |  | GGTGACAAGGCTCCACATCT |
| hsa_circ_0136505-F |  | TTGAGGACGCAGGGGAGTAT |
| hsa_circ_0136505-R |  | GGAAGGACTCCACTTCCACA |
| CXCR4-F |  | CCACGCCACCAACAGTCAGAG |
| CXCR4-R |  | CTTCTGGTGGCCCTTGGAGTG |
| GAPDH-F |  | GGGGCTCTCCAGAACATCATCC |
| GAPDH-R |  | ACGCCTGCTTCACCACCTCTT |
| U6-F |  | CTCGCTTCGGCAGCACA |
| U6-R |  | AACGCTTCACGAATTTGCGT |
| FGFR1-F |  | CCCGTAGCTCCATATTGGACA |
| FGFR1-R |  | TTTGCCATTTTTCAACCAGCG |
| hsa-miR-224-5p | miRNA | UCAAGUCACUAGUGGUUCCGUUUAG |
| Sg-CXCR4 | sgRNA | CAACCACCCACAAGTCAT |
| Sg-has-miR-224-5p | sgRNA | ATTGTGCATTGTTTCAAAA |
