# Supplementary figures and images for: Circular RNA circFGFR1 Functions as an Oncogene in Glioblastoma Cells through Sponging to hsa-miR-224-5p
Source: J Immunol Res. 2022 Jan 10;2022:7990251. doi: 10.1155/2022/7990251 (PMC8764274; doi:10.1155/2022/7990251)

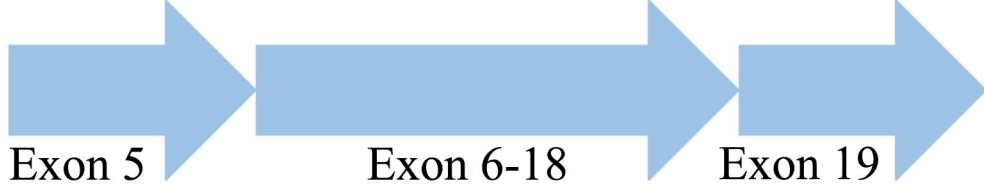

Exon 19      Exon 5  
**AACCAG** | **CCGTGA**

Back-splice site

Exon 19      Exon 5

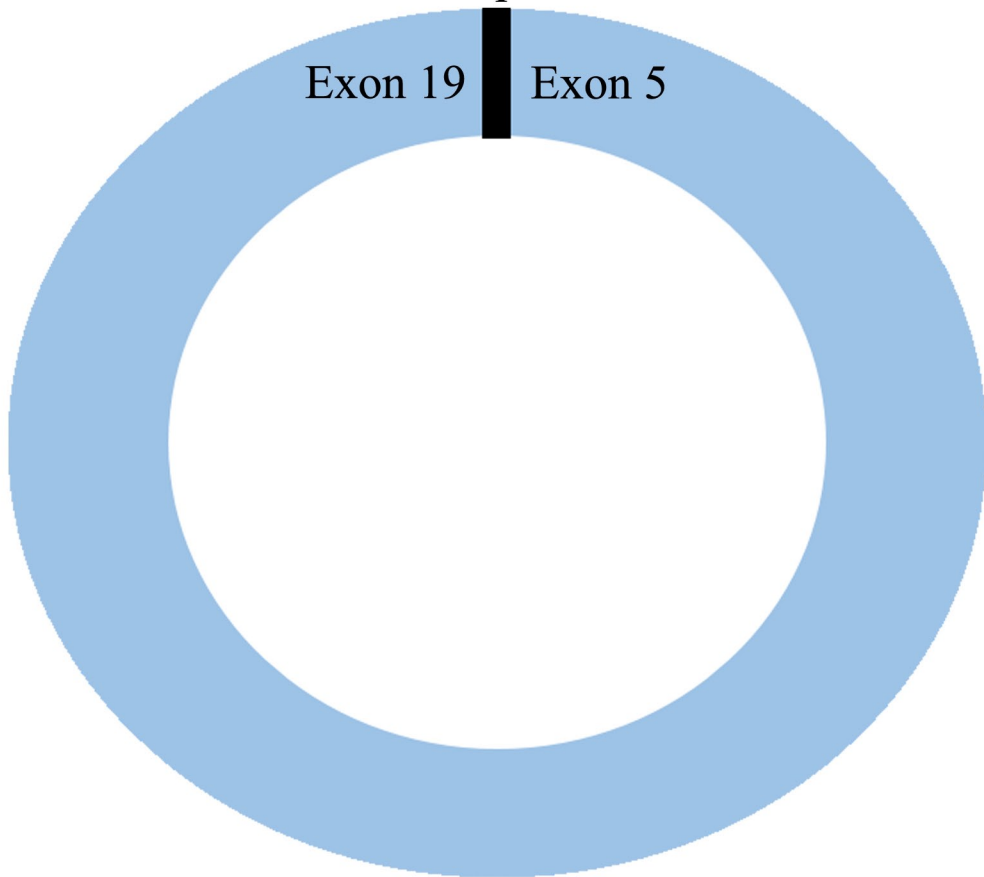

Supplement: Supplementary 2 — Figure S1: the scheme illustrating the production of circFGFR1. [file 7990251.f2.pdf]
